# Implementation of community-based management of severe acute malnutrition in conflict affected regions: a case of South Kordofan, Sudan

**DOI:** 10.1186/s13690-023-01060-z

**Published:** 2023-03-29

**Authors:** Quraish Sserwanja, Osman Omer Adam, Eissa Hassan Mohamed, Mohammed Bashir Adam, Linet M. Mutisya

**Affiliations:** 1 Programmes Department, GOAL Global, 65 House No. 227, Khartoum, Sudan; 2 Programmes Department, MSF, Khartoum, Sudan; 3 Maternal and Child Health Project, Swedish Organization for Global Health, Mayuge, Uganda

**Keywords:** Malnutrition, CMAM, OTP, Conflict- affected, Community-based management

## Abstract

Malnutrition is the major cause of mortality and morbidity globally with undernutrition contributing about 45% of all deaths of under five children. Besides the direct effects of protracted conflicts, the macroeconomic crisis that has greatly increased the national inflation rate hence devastating the purchasing power, the COVID-19 outbreak, flooding, and the Desert Locusts have contributed to a food security emergency. Besides being among the most under resourced states, South Kordofan has experienced years of conflict resulting in displacement of people and extensive infrastructure destruction with high rates of malnutrition. The state currently has 230 health facilities and out of these, only 140 are providing outpatient therapeutic programme centres with 28.6% (40) of these being operated by the state ministry of health and the rest by the international non-governmental organizations. Limited resources leading to donor dependence, limited accessibility due to insecurity and floods, poor referral system and gaps in continuity of care, lack of operational and implementation research data and limited integration of management of malnutrition in other health services have negatively affected effective implementation. Ensuring effective and efficient community based management of acute malnutrition, implementation needs action beyond the health sector with a multi-sectoral and integration approach. Federal and state development frameworks should ensure a comprehensive multi-sectoral nutrition policy with strong political commitment and allocation of adequate resources to ensure integrated and quality implementation.

## Background

Malnutrition is the major cause of mortality and morbidity globally with undernutrition contributing about 45% of all deaths of under five children [1–3]. These deaths are mainly experienced in low- and middle-income countries [1, 3] which are also experiencing increase in cases of overweight and obesity [4, 5]. Globally, about 47 million (6.9%) under five children are wasted, 144 million (21.3%) are stunted, and 38.3 million (5.6%) are overweight or obese [1].

Sudan is located in Northeast Africa with a long history of protracted social conflicts that have had a negative impact on the country’s general health and nutritional status [1, 6]. Besides the direct effects of protracted conflicts, the macroeconomic crisis has greatly increased the national inflation rate hence devastating the purchasing power. The COVID-19 outbreak, flooding, and the Desert Locusts have contributed to a food security emergency leaving 16% (7.1 million) of the population in a crisis phase and above ( phase 3 and 4) [1]. While Sustainable Development Goal 2.2 aims to end all forms of malnutrition by 2030 and achieve internationally agreed targets related to malnutrition in children [7], data on malnutrition trends in Sudan is alarming [1]. According to the 2020 global nutrition report, Sudan has made no progress towards achieving the target for stunting and wasting, with 38.2% and 16.3% of children under 5 years of age affected with stunting and wasting respectively. These rates are all higher than the average for the Africa region [8]. In about 8.6 million people lacking adequate healthcare, 3.3 million are acutely malnourished, with over half a million children suffering from severe acute malnutrition (SAM) and 2.2 million children requiring treatment for moderate acute malnutrition (MAM) [1].

In recent years, due to limited inpatient capacity, high cost of inpatient management, few trained staff resulting in low coverage, low recovery rate, high mortality, and high default rate at the inpatient therapeutic programs, the management of acute malnutrition has shifted from the traditional facility-based approach to a combination of facility and community- based management approach to improve its coverage and impact [2, 9]. This approach, known as community-based management of acute malnutrition (CMAM) consists of four key elements that include: community mobilization, outpatient supplementary feeding programme (SFP) for patients with MAM but with no medical complication, outpatient therapeutic programme (OTP) care for uncomplicated cases of SAM, and stabilization center / inpatient care to SAM patients with medical complications [9]. CMAM enabling factors include; use of a simple screening tool (MUAC tape ) to detect both moderate and severe acute malnutrition by measuring the mid-upper arm circumference, home treatment using ready-to-use therapeutic foods (RUTF) and routine medical checkups, and use of a simple classification system that distinguishes SAM patient with medical complications from those with no medical complications [7]. Existing CMAM programmes have documented success with improved recovery and mortality rates [2, 7].

South Kordofan population is estimated to be 2.5 million people and is located in the South Central region of Sudan bordering South Sudan to the South [6]. The state has experienced years of conflict resulting in displacement of over 200 000 people and extensive destruction of infrastructure. [6]. It’s among the most under resourced states, which greatly limits essential service delivery [6]. The global prevalence of underweight and wasting among children under five years in this regions is 26.1% and 7.8% respectively[1].

## Current status of CMAM implementation in South Kordofan state

Health and nutrition services are under the mandate of the South Kordofan state ministry of health (SMOH) which is oversighted by the Khartoum federal ministry of health (FMOH). South Kordofan has 14 localities in which the health and nutrition services are directly implemented by the locality health departments (LHDs) with the support of various international non-government organizations [6, 10, 11]. The state currently has 230 health facilities and out of these, only 140 are providing outpatient therapeutic programme (OTP) for SAM cases without medical complications. Out of these 140 health facilities, 28.6% (40) are operated by SMOH and the rest by international non-governmental organizations (INGOs). In addition, out of the 140 OTP centres, 73.6% (103) are currently providing targeted supplementary feeding programme (TSFP) for the management of MAM. Out of the 103 TSFP centers, 41.7% (43) are operated by SMOH while the rest by the INGOs. Out of the 14 localities in the state, only 11 have stabilization centres for the management of SAM cases with medical complications and only 63.6% (7) of these are operated by SMOH with the rest being operated by the INGOs. Community mobilization activities and screenings are done mainly through the support of the various INGOs.

## Challenges and recommendations

Table 1 below summarizes CMAM implementation challenges and recommendations in South Kordofan, Sudan.

**Table 1:** CMAM implementation challenges and recommendations in South Kordofan, Sudan

Sn	Challenges	Suggested recommendations
1.	**INGO dependency and limited SMOH financial resources.** The state mainly depends on INGOs to operate most the OTP and TSFP centres by providing financial & technical support and project coordination, which affects sustainability of the programme after INGOs’ hand over.	The SMOH may use advocacy to lobby for allocation of more resources for nutrition programs. SMOH should focus on programme-based budgeting which attempts to allocate expenditures by program. The focus should be directed to preventive services which is underfinanced.
2.	**Shortage /low motivated Nutrition officers** Due to low salaries offered by the state and INGOs, nutritionists are demotivated to work in OTP and TSFP centres. For example, The SMOH nutritionists earn between 27 and 40 United States Dollars (USD) per month and volunteers earn between 16 and 24 USD per month which is not very different from what INGOs offer. The salaries are sometimes paid out late. The demotivated staff end up working less hours which affects service delivery. These conditions discourage qualified nutritionists from seeking the open positions, leaving volunteers to run the projects.	The National government should focus more on strengthening Human Resources for Health (HRH) to address the staffing challenges (limited resources, maldistribution of nutrition officers, poor retention of nutrition officers in the rural areas) experienced in the region.
3.	**Insecurity** Some localities such as Abujubaiha, Talodi, Rashad and AbuKarshola experience periodic tribal conflicts which tend to impede movement to and within these localities. This disrupts delivery of nutrition and other health services.	It would be a noble idea to integrate conflict perspectives in design stage of programs and projects with aim to minimize negative and maximize positive contributions to conflict prevention and peace building.
4.	**Floods** Heavy rainfall seasons render roads impassable, cutting off transportation of nutrition commodities. This leads to shortage and sometimes stock out of both medical and nutrition supplies at OTP, TSFP and stabilization centres. Program monitoring is also affected as reporting and support supervision by SMOH is not conducted. Community based activities such as mobilization and mass screening are negatively affected, registering a low turn out for case identification.	Construction of large warehouse and/or expansion of storage spaces within the health facilities is crucial to ensure pre-positioning of supplies to avoid stock outs during the rainy season. For future considerations, the state would initiate in-country production of RUTF using locally available ingredients which is important for ensuring cost effective readily available supplies to manage severe acute malnutrition. This will ensure sustainable CMAM programming.
5.	**Poor referral system and gaps in continuity of care** Limited availability of ambulances coupled by poor road networks are a major challenge leading to high transport costs and longer hours spent on the road. This has a negative impact on emergency cases that need referral to the stabilization centres with some cases reaching the referral centres when it is too late to be saved. Some children referred to TSFP upon discharged from OTP do not join due to lack of transport to the nearest TSFP centre. This affects continuity of care.	The state should endeavor to avail stabilization centers in each locality and support all health facilities to provide OTP, TSFP services. The state should also strengthen the referral system for efficient and effective delivery of health services.
6.	**Lack of operational and implementation research data** The different INGOs work with the oversight of the SMOH but in isolation with no clear way of sharing data and lessons learnt with each other. The lack of readily available operational research data limits evidence-based implementation. In South Kordofan and similar contexts, timely and accurate data collection is affected by poor internet access, limited phone network coverage, poor roads and limited availability of trained staff [6]. Delayed and less accurate reporting affect evidence-based decision making and planning.	To ensure effective and well-coordinated CMAM implementation, the different stakeholders need to have the right to information via health information systems and data platforms. Investing in infrastructure development such as roads and telecommunication will improve timely reporting and increased incentives for trained staff working in hard-to-reach areas. Periodic refresher trainings on data management will ensure accurate and timely reporting for efficient planning. There is need for INGOs and the SMOH to initiate an operational research initiative for continuous project evaluation. It would also be valuable to allocate resources to study the implementation of CMAM (implementation research) seeking to understand further the real implementation conditions in South Kordofan.
	**Limited integration of nutrition services in other health services** Limited availability of enough and trained staff has negatively affected integration of nutrition services such as MUAC screening and nutrition counselling at the triage, outpatient department (OPD) consultation rooms and during vaccination days. This leads to less timely identification of cases leading to delayed diagnoses that are usually severe. Furthermore, lack of joint state cluster coordination meetings for nutrition, WASH and health makes it difficult to share lessons learnt and best practices across the different sectors for replication.	The on-job and classroom trainings for all health care workers (to include all cadres) should be revised to ensure that nutrition topics such as MUAC screening and infant and young child feeding (IYCF) practices are incorporated in order to improve knowledge, skills and be in a position to integrate and/or provide nutrition services alongside other health care services offered to children, pregnant and lactating women (PLW). There is need to introduce IYCF ‘corners’ in health facilities and increase IYCF awareness through increased facilitation of community nutrition volunteers. SMOH should establish a joint learning and sharing fora where stakeholders and health departments meet, report, and share information, lesson learnt and best practices.

## Conclusion

To ensue effective and efficient CMAM implementation, multi-sectoral integration is paramount. Federal and state development frameworks should ensure a comprehensive multi-sectoral nutrition policy with strong political commitment and allocation of adequate resources to ensure integrated and quality CMAM implementation. Community based mechanisms ensuring growth monitoring activities and routine nutritional screening activities should be strengthened to increase access and utilization. Planning, advocating and lobbying for sustainable SMOH funding of the CMAM activities should be prioritized in order to minimize overdependence on donors and INGOs.

## Data Availability

Not applicable.

## Abbreviations

WHO: World Health Organization
SAM: Severe Acute Malnutrition
CMAM: Community-based Management of Acute Malnutrition
MAM: Moderate Malnutrition
OTP: Outpatient Therapeutic Programme
TSFP: Targeted Supplementary Feeding Programme
RUTF: Ready to Use Therapeutic Feeds
FMoH: Federal Ministry of Health
SMoH: State Ministry of Health
NGO: Non-Governmental Organizations
PLW: Pregnant and Lactating Women
